# Assessment of the Association Between Coronary Artery Calcification, Plaque Vulnerability, and Perivascular Inflammation via Coronary CT Angiography

**DOI:** 10.3390/life15081288

**Published:** 2025-08-13

**Authors:** Botond Barna Mátyás, Imre Benedek, Nóra Rat, Emanuel Blîndu, Ioana Patricia Rodean, Ioana Haja, Delia Păcurar, Theofana Mihăilă, Theodora Benedek

**Affiliations:** 1 Clinic of Cardiology, Mureș County Emergency Clinical Hospital, 540136 Târgu Mureș, Romania; matyas_botond@yahoo.com (B.B.M.); imre.benedek@umfst.ro (I.B.); emi.blindu@yahoo.com (E.B.); ioana.rodean@umfst.ro (I.P.R.); ioana.haja94@gmail.com (I.H.); deliapacurar99@gmail.com (D.P.); theofana_m@yahoo.com (T.M.); theodora.benedek@umfst.ro (T.B.); 2 Doctoral School of Medicine and Pharmacy, “George Emil Palade” University of Medicine, Pharmacy, Science and Technology of Târgu Mureș, 540139 Târgu Mureș, Romania; 3 Center of Advanced Research in Multimodality Cardiac Imaging, CardioMed Medical Center, 540124 Târgu Mures, Romania; 4 Department of Cardiology, “George Emil Palade” University of Medicine, Pharmacy, Science and Technology of Târgu Mureș, 540139 Târgu Mureș, Romania

**Keywords:** coronary artery disease, coronary artery calcium score, coronary computed tomography angiography, CAD-RADS, high-risk plaque features, perivascular inflammation, fat attenuation index

## Abstract

Background: Coronary artery calcium (CAC) scores are a widely used surrogate marker for atherosclerotic burden, but they do not fully reflect plaque vulnerability or coronary inflammation. This study aimed to evaluate the relationship between CACs, coronary plaque characteristics, and perivascular inflammatory activity using advanced CCTA and CaRi-Heart^®^ analysis. Methods: A total of 250 patients with no prior cardiovascular disease were retrospectively evaluated and stratified by CACs into three groups: 0 (*n* = 28), 1–100 (*n* = 121), and >100 (*n* = 101). Coronary plaque morphology, high-risk plaque (HRP) features, CAD-RADS scores, and AI-derived fat attenuation index (FAI) centiles were assessed. Results: Significant differences across CAC categories were observed for several key parameters. The number of diseased coronary segments increased markedly (from 1.39 ± 1.10 vs. 2.97 ± 1.57 vs. 3.94 ± 2.10; *p* < 0.0001, one-way ANOVA). A similar upward trend was seen for segment involvement scores, HRP prevalence, and the proportions of mixed and calcified plaque components. Regression analysis demonstrated that CACs correlated significantly with segment burden (r^2^ = 0.2520), CAD-RADS (r^2^ = 0.1352), and the FAI score centile (r^2^ = 0.0568). Conclusions: This study highlights the limitations of CACs as a standalone risk stratification tool. Vulnerable and inflamed plaques may already be present in patients with low or zero CACs. Integrating CCTA with perivascular FAI mapping enables earlier detection of biologically active atherosclerosis and supports more precise clinical decision-making.

## 1. Introduction

CAD is the leading global cause of death and disability, predominantly driven by the rupture of vulnerable atherosclerotic plaques and the subsequent thrombotic occlusion of coronary arteries [1,2]. Coronary computed tomography angiography (CCTA), complemented by coronary artery calcium scores (CACs), has emerged as a cornerstone in the non-invasive evaluation of CAD, enabling detailed assessment of coronary lumen stenosis and plaque morphology [3,4,5]. However, traditional calcium scoring often fails to identify biologically active, lipid-rich, non-calcified plaques, particularly in younger or intermediate-risk individuals with low or zero CACs [6]. This diagnostic blind spot is significant, as numerous ACS originate from such lesions in patients deemed low risk by calcium scoring alone [7].

A growing body of evidence suggests that atherosclerosis involves both structural and metabolic–inflammatory mechanisms. Bioactive lipids like ceramides and sphingosine-1-phosphate contribute to endothelial dysfunction and plaque vulnerability, often preceding detectable calcification [8]. Recent studies also highlight the role of early immune activation and oxidative stress in subclinical disease progression, particularly in low-risk populations. In parallel, emerging therapies such as the PCSK9 inhibitor E28362 have demonstrated lipid-lowering and anti-atherosclerotic effects in experimental models, underscoring the clinical value of identifying high-risk, inflammation-driven lesions before overt calcification develops [9,10].

Recent advancements have focused on the perivascular adipose tissue fat attenuation index (PVAT-FAI), a novel imaging biomarker of localized coronary inflammation that reflects cytokine-mediated changes in the composition of perivascular adipose tissue [11,12]. FAI mapping allows for the identification of early vascular inflammation that precedes plaque rupture and predicts future adverse cardiovascular events independent of stenosis severity [13]. Studies such as CRISP-CT and ORFAN have demonstrated the clinical utility of FAI, showing that elevated values are strongly associated with high-risk plaque (HRP) features—such as low-attenuation cores (LAPs), positive remodeling (PR), and napkin-ring signs (NRSs)—and are predictive of MACE [14,15,16]. Furthermore, artificial intelligence-enabled platforms like the CaRi-Heart^®^ system provide automated, high-throughput radiomic analysis that adjusts for individual and scan-level variability, increasing the reliability of FAI measurements across imaging centers [14,17].

Despite these developments, most FAI-based studies have focused on high-risk populations with advanced CAD, limiting their generalizability. There remains a paucity of data on perivascular inflammation in patients with a low-to-intermediate pre-test probability of CAD, especially those with minimal or no coronary calcification [18]. Importantly, even in such populations, several case-based reports and retrospective studies have highlighted the presence of inflamed, non-calcified plaques capable of causing significant stenosis and adverse cardiac outcomes [19,20]. This highlights the need to integrate biological indicators of inflammation with anatomical assessment in CCTA, particularly in patients with ambiguous or low-risk calcium scores.

While prior studies—such as CRISP-CT—have explored FAI in relation to outcomes and plaque burden, few have examined its association with CACs, particularly in asymptomatic, low-risk populations. Our study addresses this gap by analyzing AI-derived FAI centiles across stratified CAC groups, offering new insights into inflammatory risk in patients with minimal or no calcification.

This study aimed to address the limitations of calcium scoring in detecting biologically active CAD by conducting a substudy within the INTEL-FAT project at the George Emil Palade University of Medicine, Pharmacy, Science, and Technology of Târgu Mureș, Romania. Leveraging CCTA combined with AI-based analysis via the CaRi-Heart^®^ platform, we evaluated perivascular inflammation through FAI mapping, alongside comprehensive plaque assessment and CAD-RADS classification. This multimodal approach allowed us to investigate the relationship between coronary plaque characteristics and local vascular inflammation, and to determine whether FAI improves the detection of high-risk, inflammation-driven plaques that may be overlooked by traditional calcium scoring.

## 2. Materials and Methods

### 2.1. Study Design and Population

This retrospective substudy was conducted at the Center for Advanced Research in Multimodality Cardiac Imaging, part of the Cardiomed Medical Center in Târgu Mureș, Romania—a specialized facility focused on cardiovascular diagnostics and translational research. The analysis is embedded within the broader INTEL-FAT study and included a cohort of 250 consecutively enrolled adult patients who underwent CCTA between January 2022 and December 2024.

During this interval, over 3500 patients underwent CCTA examinations at our center, encompassing a heterogeneous population ranging from asymptomatic individuals to those with known CAD or prior myocardial infarction (MI). CCTA referrals were typically based on clinical symptoms (e.g., atypical chest pain), abnormal ECG findings, inconclusive echocardiography, or the presence of multiple cardiovascular risk factors. All patients included in this substudy presented with a low-to-intermediate pre-test clinical probability of CAD, in accordance with contemporary ESC guidelines, and were referred for diagnostic imaging as part of a primary prevention strategy [21].

Eligible patients were aged between 18 and 69 years and had no prior history of CVD, including CAD, MI, heart failure, stroke, or peripheral arterial disease. Individuals with diabetes mellitus (DM) were also excluded to minimize potential confounding effects on vascular inflammation and plaque characteristics. Only CCTA studies with diagnostic image quality were included. The study cohort was systematically stratified into three distinct groups based on CACs, which served as a surrogate marker of total coronary atherosclerotic burden. Specifically, Group 1 consisted of individuals with no detectable coronary calcium (CACs = 0, *n* = 28); Group 2 included those with mild-to-moderate calcification (CACs 1–100, *n* = 121); and Group 3 comprised patients with a high calcium burden (CACs > 100, *n* = 101). This stratification enabled a comparative analysis of anatomical, morphological, and inflammatory CCTA-derived parameters across a spectrum of coronary calcification levels in patients without established cardiovascular disease.

Participants meeting any of the following criteria were not included in this study: (1) a history of coronary revascularization procedures, including PCI or CABG, performed prior to CCTA; (2) documented ACS occurring before the imaging assessment; (3) previously diagnosed CAD or any other form of established CVD, such as MI, heart failure, or cerebrovascular events; (4) a known history of DM, due to its independent effects on vascular inflammation and atherosclerotic remodeling; and (5) suboptimal image quality rendering the CCTA non-diagnostic, including cases affected by severe motion artifacts, extensive blooming from heavy calcifications, or other technical limitations that impaired accurate plaque characterization.

**Figure 1:**
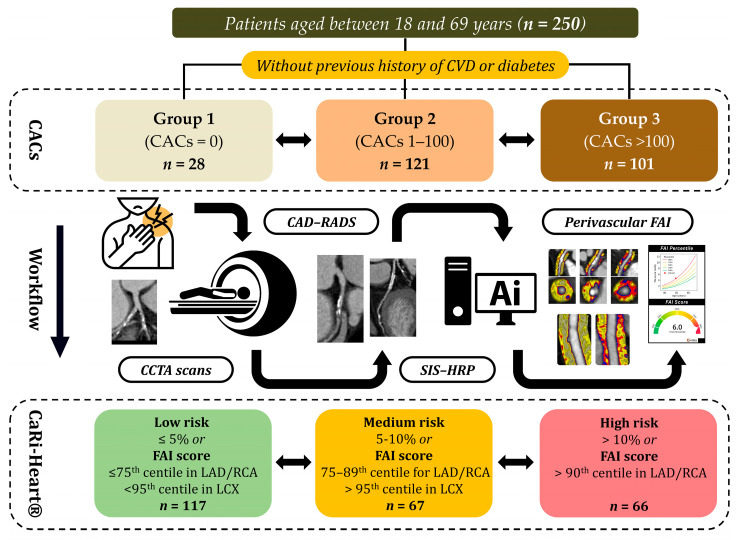
Schematic representation of the study protocol. Abbreviations: CVD—cardiovascular disease; CACs—coronary artery calcium scores; CCTA—coronary computed tomography angiography; CAD-RADS—coronary artery disease-reporting and data system; SIS—segment involvement score; HRP—high-risk plaque; FAI—fat attenuation index; LAD—left anterior descending artery; RCA—right coronary artery; LCX—left circumflex artery.

The overall study design, including patient selection criteria, grouping according to coronary artery calcium scores, imaging workflow, and risk stratification based on CAD-RADS, SIS, HRP, and perivascular FAI analysis, is presented in Figure 1.

### 2.2. CCTA Acquisition Protocol and Image Post-Processing Workflow

CCTA was conducted using a 128-slice CT system (Somatom Definition AS, Siemens Healthcare, Erlangen, Germany) at the Center for Advanced Research in Multimodality Cardiac Imaging, Cardiomed Medical Center. A retrospective ECG-gated acquisition protocol was employed, specifically tailored for patients with heart rates below 65 bpm. When necessary, oral or intravenous beta-blockers were administered to achieve optimal heart rate control, with continuous monitoring of blood pressure throughout the procedure. The imaging protocol began with a non-contrast scan for CACs, followed by a contrast-enhanced acquisition. This involved the administration of 80–100 mL of an iodine-based contrast agent, immediately followed by a 50 mL saline chaser at an injection rate of 5.5–6 mL/s during a single breath-hold. Scanning parameters included a tube voltage of 120 kV, a gantry rotation time of 0.33 s, and detector collimation of 128 × 0.6 mm. All image data were stored digitally for subsequent analysis and post-processing.

Following image acquisition, all datasets were anonymized, converted to DICOM format, and securely transferred to Caristo Diagnostics (Oxford, UK) for advanced radiomic analysis. Quantitative assessment of PVAT-FAI and coronary plaque morphology was performed using the CaRi-Heart^®^ platform. Automated segmentation and high-resolution 3D radiomic quantification of perivascular adipose tissue were applied to all major coronary vessels. This AI-powered method corrects for scan variability and enables accurate measurement of vascular inflammation. CaRi-Heart^®^ uses a proprietary cloud-based algorithm to analyze perivascular fat attenuation, adjusted for patient age, sex, and vessel location. FAI centile scores are benchmarked against large normative datasets, allowing for the classification of patients into low-, intermediate-, or high-risk inflammation categories [20,22].

CaRi-Heart^®^ risk scores were compared across three predefined CACs groups to assess differences in inflammation levels, plaque types, and plaque vulnerability. Based on the analysis of each patient’s coronary vessels, individuals were assigned to a low-, intermediate-, or high-perivascular-inflammation-risk group, regardless of how many vessels were evaluated.

Anatomical and morphological plaque analysis was conducted across all affected coronary segments (*n* = 797). For each patient, a segment involvement score (SIS) was calculated (mean ± SD: 3.18 ± 1.93), and individuals were classified into three groups based on the number of diseased segments: ≤2, 3–4, or >4. Plaque composition was categorized as non-calcified, mixed, or calcified. HRP features were identified according to standardized CT criteria and included LAP, PR, SC, and the NRS. HRP prevalence was defined as the presence of at least one such feature in any coronary segment per patient. To further stratify disease severity, the CAD-RADS scoring system was used, categorizing findings into three groups: minimal or no stenosis (scores 0–1), moderate stenosis (scores 2–3), and severe stenosis (scores 4–5).

### 2.3. Statistical Analysis

Upon receiving the inflammatory dataset from our collaborators in Oxford, the data were systematically organized using Microsoft Excel (Microsoft Corporation, Redmond, WA, USA) for subsequent statistical evaluation. All analyses were performed using GraphPad Prism version 10.3.1 (GraphPad Software, Inc., San Diego, CA, USA). Continuous variables were expressed as mean ± SD. Overall group differences were assessed using one-way analysis of variance (ANOVA), followed by Tukey’s honestly significant difference (HSD) post hoc test for pairwise comparisons. Categorical variables were summarized as absolute numbers and percentages, with overall comparisons conducted via the chi-square (χ^2^) test. For binary or small-sample comparisons, Fisher’s exact test was applied. Bonferroni correction was used for multiple pairwise comparisons of categorical variables, with an adjusted alpha level of 0.0167. Additionally, linear regression models were employed to explore associations between CACs and key anatomical or inflammatory markers, including the number of affected coronary segments, CAD-RADS grading, and perivascular FAI centile scores. A threshold of *p* < 0.05 was used to determine statistical significance.

## 3. Results

### 3.1. Baseline Demographics and Clinical Characteristics of the Study Population

A total of 250 patients were included in this study, with a mean age of 61.1 ± 6.3 years and a predominance of male participants (74.0%). Based on CACs, participants were stratified into three groups: Group 1 (CACs = 0, *n* = 28), Group 2 (CACs 1–100, *n* = 121), and Group 3 (CACs > 100, *n* = 101). A statistically significant trend in increasing age was observed across the groups, from 55.9 ± 7.1 years in Group 1 to 63.4 ± 4.8 years in Group 3, with significant differences between Group 1 and Group 2 (*p* = 0.0011), Group 2 and Group 3 (*p* = 0.0004), and Group 1 and Group 3 (*p* < 0.0001), and across all groups (one-way ANOVA, *p* ≤ 0.0001).

Body mass index (BMI) also increased progressively across the CAC categories, with mean values of 25.05 ± 3.5 kg/m^2^ in Group 1, 27.93 ± 3.9 kg/m^2^ in Group 2, and 28.07 ± 3.4 kg/m^2^ in Group 3. One-way ANOVA confirmed a significant difference in BMI across the three groups (*p* = 0.0005). Pairwise comparisons demonstrated a significant difference between Group 1 and Group 2 (*p* = 0.0008), and between Group 1 and Group 3 (*p* = 0.0005), whereas no significant difference was observed between Group 2 and Group 3 (*p* = 0.9589), suggesting that the primary shift in BMI occurs between patients without detectable calcification and those with any measurable CACs. In contrast, no significant differences were found in left ventricular ejection fraction (LVEF) or gender distribution across the three groups (one-way ANOVA, *p* = 0.6613 and *p* = 0.1285, respectively).

The prevalence of hypertension increased markedly with rising CACs, with 32.14% in Group 1, 58.68% in Group 2, and 69.31% in Group 3. A statistically significant difference was noted between Group 1 and Group 2 (*p* = 0.0376), while the difference between Group 2 and Group 3 was not significant (*p* = 0.3710). A significant difference was also observed between Group 1 and Group 3 (*p* = 0.0023). The overall trend across the three groups was significant according to overall χ^2^ test (*p* = 0.0017), suggesting a progressive increase in hypertension prevalence with increasing CACs. A comparable pattern was seen for hypercholesterolemia, with prevalence rates of 25.00%, 57.02%, and 65.35% across Groups 1 to 3, respectively. A significant difference was observed between Group 1 and Group 2 (*p* = 0.0089), while the comparison between Groups 2 and 3 did not reach significance (*p* = 0.6513). Group 1 vs. Group 3 also showed a significant difference (*p* = 0.0006). The overall group comparison via overall χ^2^ test was significant (*p* = 0.0007), supporting a relationship between lipid abnormalities and CAC severity. Conversely, no statistically significant differences were found between groups for smoking status, obesity, or family history of CAD, as all pairwise and global p-values were above the threshold for significance, indicating a relatively uniform distribution of these risk factors across CAC categories.

When applying the SCORE2 algorithm for cardiovascular risk estimation, a higher proportion of patients in Group 1 were classified as low-to-moderate risk (57.14%) compared to Group 2 (38.84%) and Group 3 (39.60%). However, the difference between Group 1 and Group 2 did not reach statistical significance (*p* = 0.2754), and no differences were observed between Group 2 and Group 3 (*p* > 0.9999) or between Group 1 and Group 3 (*p* = 0.3939), nor in the overall comparison across all groups (overall χ^2^ test, *p* = 0.1901). Similarly, the distribution of patients in the high (overall χ^2^ test, *p* = 0.6567) and very high (overall χ^2^ test, *p* = 0.3941) SCORE2 risk categories did not significantly differ among the CAC groups. Nevertheless, the general pattern suggests a shift toward higher cardiovascular risk classifications as CACs increase, despite the lack of statistically significant differences in the pairwise and overall comparisons.

A comprehensive summary of baseline clinical characteristics, cardiovascular risk profiles, and SCORE2 classification by CAC category is presented in Table 1.

**Figure 2:**
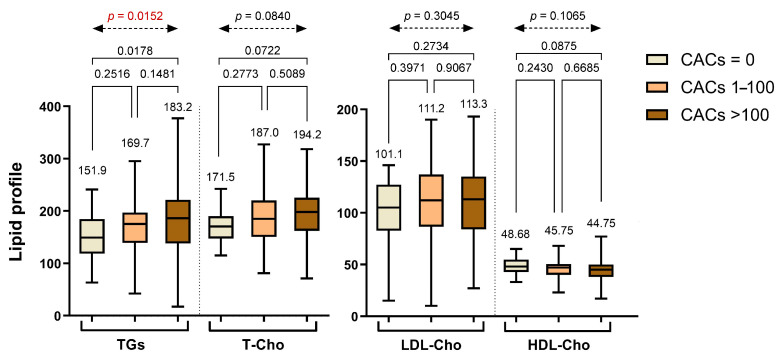
Lipid profile parameters stratified across different CAC groups. Abbreviations: TGs—triglycerides; T-Cho—total cholesterol; LDL-Cho—low-density lipoprotein cholesterol; HDL-Cho—high-density lipoprotein cholesterol.

Regarding lipid profiles, triglyceride (TG) levels showed a progressive increase across the CAC categories, with mean values rising from Group 1 to Group 3. Although the pairwise comparisons between Group 1 and Group 2 (*p* = 0.2516) and between Group 2 and Group 3 (*p* = 0.1481) did not reach statistical significance individually, the overall difference across the three groups was statistically significant based on one-way ANOVA (*p* = 0.0152). Additionally, a significant difference was observed between Group 1 and Group 3 (*p* = 0.0178), suggesting a general upward trend in TG levels with increasing CACs, particularly between patients with no calcification and those with a high calcium burden. No significant differences were found in total cholesterol (T-Cho), low-density lipoprotein cholesterol (LDL-Cho), or high-density lipoprotein cholesterol (HDL-Cho) across the CAC categories, as none of the pairwise or overall comparisons reached significance. All lipid parameters across the groups are summarized in Figure 2.

### 3.2. Coronary Plaque Distribution, Composition, and Inflammatory Characteristics According to CAC Groups

**Figure 3:**
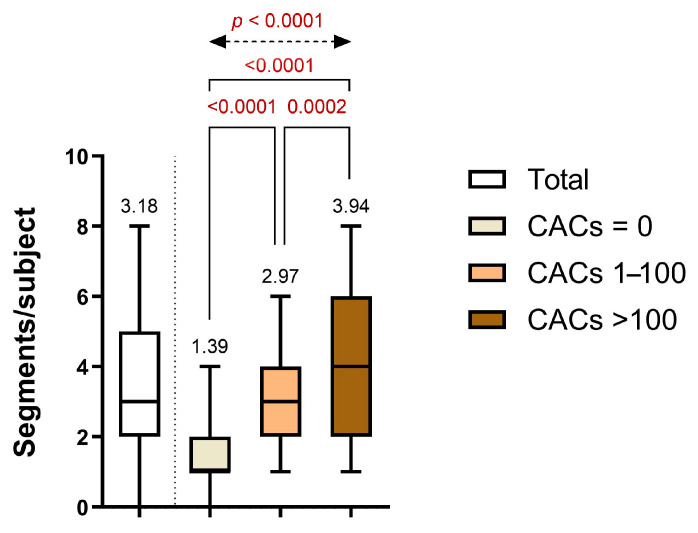
Distribution of the diseased coronary segments per subject across CAC categories.

Segmental analysis of CCTA demonstrated a clear escalation in atherosclerotic burden across increasing CAC groups. The average number of affected coronary segments per patient increased significantly from 1.39 ± 1.10 in Group 1 (CACs = 0) to 2.97 ± 1.57 in Group 2 (CACs 1–100), and further to 3.94 ± 2.10 in Group 3 (CACs > 100). Statistically significant differences were observed between Group 1 and Group 2 (*p* = 0.0002), between Group 1 and Group 3 (*p* < 0.0001), and between Group 2 and Group 3 (*p* < 0.0001), with an overall significant difference confirmed by one-way ANOVA (*p* < 0.0001) (Figure 3).

**Figure 4:**
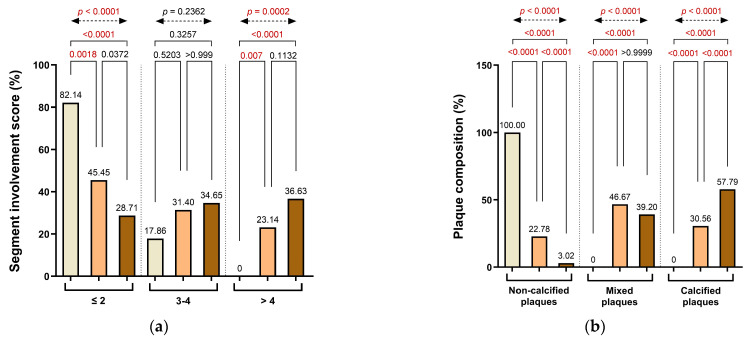

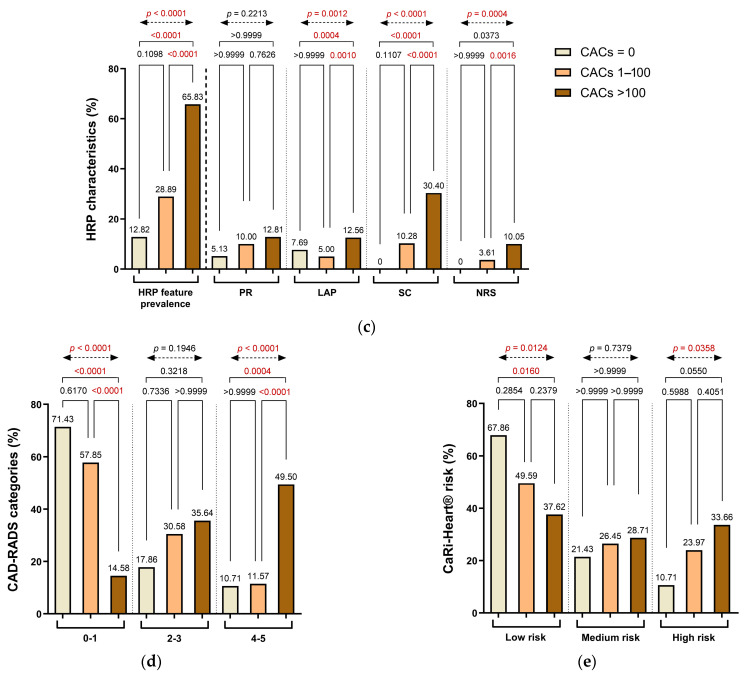
Distribution of (**a**) segment involvement, (**b**) plaque composition, (**c**) high-risk plaque (HRP) features, (**d**) CAD-RADS categories, and (**e**) CaRi-Heart^®^ inflammatory risk across CAC groups. Abbreviations: HRP—high-risk plaque; PR—positive remodeling; LAP—low-attenuation plaque; SC—spotty calcification; NRS—napkin-ring signs; CAD-RADS—Coronary Artery Disease-Reporting and Data System.

A similar pattern was reflected in the distribution of the SIS. The majority of patients in Group 1 had limited disease involvement (≤2 involved segments: 82.14%), whereas this proportion declined in Groups 2 (45.45%) and 3 (28.71%). In contrast, patients with extensive disease involvement (>4 affected segments) were observed exclusively in Groups 2 and 3 (23.14% and 36.63%, respectively). These differences were statistically significant between Group 1 and Group 2 (*p* = 0.0018), between Group 1 and Group 3 (*p* < 0.0001), and between Group 2 and Group 3 (*p* = 0.0070), with a robust overall significance across all groups (*p* < 0.0001). Involvement of 3–4 coronary segments was infrequent in Group 1 (17.86%) but became more prevalent in Group 2 (31.40%) and Group 3 (34.65%), reflecting a gradual escalation in disease complexity. These findings underscore the progressive nature of anatomical disease burden with increasing CACs and highlight Group 2 as a transitional stage, characterized by a heterogeneous distribution of both moderate and extensive segmental involvement (Figure 4a).

Plaque morphology demonstrated distinct variation across CACs categories. Non-calcified plaques were exclusively observed in Group 1 (100%) and decreased sharply in Groups 2 and 3, accounting for only 22.78% and 3.02% of plaques, respectively. The decline in non-calcified plaque prevalence was statistically significant in both pairwise comparisons (Group 1 vs. Group 2 and Group 2 vs. Group 3: *p* < 0.0001), as well as between Group 1 and Group 3 (*p* < 0.0001) and across all groups (overall χ^2^ test, *p* < 0.0001). Conversely, mixed plaques were absent in Group 1, but frequently detected in Group 2 (46.67%) and Group 3 (39.20%), reflecting a transition toward more heterogeneous plaque composition with increasing calcium burden. These differences were statistically significant in the overall comparison (overall χ^2^ test, *p* < 0.0001), with no significant differences between adjacent groups. Calcified plaques followed a similar progression, increasing from 30.56% in Group 2 to 57.79% in Group 3, with strong statistical significance observed between all group comparisons (*p* < 0.0001), including Group 1 vs. Group 3 (*p* < 0.0001), underscoring the shift toward more advanced plaque phenotypes in patients with elevated CACs (Figure 4b).

HRP characteristics were also more frequently encountered in patients with greater CACs. The prevalence of HRP features rose from 12.82% in Group 1 to 28.99% in Group 2 and 65.83% in Group 3. Although the difference between Group 1 and Group 2 was not statistically significant (*p* = 0.1098), significant differences were observed between Group 2 and Group 3 (*p* < 0.0001), between Group 1 and Group 3 (*p* < 0.0001), and in the overall analysis (overall χ^2^ test, *p* < 0.0001), reflecting a progressive increase in plaque vulnerability.

Among individual HRP components, spotty calcification (SC) showed a pronounced increase with rising CAC severity, being absent in Group 1 and present in 10.28% and 30.40% of patients in Groups 2 and 3, respectively. These differences were statistically significant between Group 2 and Group 3 (*p* < 0.0001), between Group 1 and Group 3 (*p* = 0.0001), and in the overall analysis (*p* < 0.0001), although the difference between Group 1 and Group 2 did not reach significance (*p* = 0.1107). In contrast, LAP decreased from 7.69% in Group 1 to 5.00% in Group 2, and then increased to 12.56% in Group 3, with statistical significance observed between Group 2 and Group 3 (*p* = 0.0004), Group 1 and Group 3 (*p* = 0.0012), and in the overall comparison (*p* < 0.0001). PR prevalence did not differ significantly among the groups. The napkin-ring sign (NRS) was not observed in Group 1 but was present in 3.61% of patients in Group 2 and 10.05% in Group 3. Although relatively uncommon, its frequency increased with CACs, with statistically significant differences between Group 2 and Group 3 (*p* = 0.0006), Group 1 and Group 3 (*p* = 0.0004), and across the entire cohort (overall χ^2^ test, *p* < 0.0001) (Figure 4c).

CAD severity, as assessed by the CAD-RADS classification, was closely associated with the extent of coronary calcification. In Group 1, most patients (71.43%) were categorized as CAD-RADS 0–1, indicating minimal or no stenosis. This proportion dropped substantially in Group 2 (57.85%) and even further in Group 3 (14.58%). Conversely, the prevalence of advanced stenosis (CAD-RADS 4–5) increased progressively, from 10.71% in Group 1 to 11.57% in Group 2 and 49.50% in Group 3. Significant differences were observed between Group 1 and Group 3 (*p* < 0.0001) and Group 2 and Group 3 (*p* < 0.0001), while comparisons between Group 1 and Group 2 were not significant. A significant overall difference was observed across all three groups (overall χ^2^ test, *p* < 0.0001), supporting a trend toward more severe CAD-RADS categories with rising CACs (Figure 4d).

Inflammatory risk stratification, determined using the CaRi-Heart^®^ platform, also showed a graded distribution across CACs categories. Low-risk inflammatory profiles were most prevalent in Group 1 (67.86%) and declined progressively in Group 2 (49.59%) and Group 3 (37.62%). In contrast, high-risk designations were observed in 10.71% of patients in Group 1, increasing to 23.97% in Group 2 and 33.66% in Group 3. Although pairwise comparisons between Groups 1 and 2 and between Groups 2 and 3 did not reach statistical significance, the differences between Group 1 and Group 3 were significant for both the low-risk (*p* = 0.0124) and high-risk (*p* = 0.0358) categories. The overall group differences were also significant for both low-risk (*p* = 0.0103) and high-risk designations (*p* = 0.0373), reflecting a meaningful shift toward elevated inflammation-related risk with rising CACs and supporting the progressive nature of atherosclerotic disease and perivascular inflammatory activity (Figure 4e).

Table 2 presents plaque composition, HRP features, and CaRi-Heart^®^ risk classification across CAC categories, showing significant intergroup differences.

**Figure 5:**
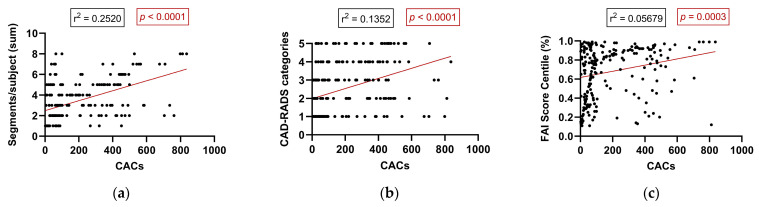
Correlation between CACs and anatomical and inflammatory parameters in patients with detectable calcification (CAC > 0): (**a**) number of diseased coronary segments, (**b**) CAD-RADS category, and (**c**) FAI score centile. Abbreviations: CAD-RADS—Coronary Artery Disease-Reporting and Data System; FAI—fat attenuation index.

In patients with detectable CACs (CAC > 0), regression analysis demonstrated a significant association between calcium burden and multiple anatomical and inflammatory parameters. A moderate positive correlation was found between CACs and the total number of diseased coronary segments per subject (r = 0.2520, *p* < 0.0001), indicating that higher CACs are associated with more extensive coronary involvement. Similarly, a statistically significant—though weaker—correlation was observed between CACs and CAD-RADS categories (r = 0.1352, *p* < 0.0001), reflecting a tendency for greater calcium burden to coincide with more advanced stenotic disease. Additionally, a weak but statistically significant correlation emerged between CACs and the perivascular FAI score centile (r = 0.0568, *p* = 0.0003), suggesting that increasing coronary calcification is modestly associated with elevated perivascular inflammation (Figure 5). Collectively, these findings support the notion that within individuals with established coronary calcification, increasing CACs are linked not only to greater anatomical plaque burden and stenosis severity but also to a gradual rise in coronary inflammation as assessed by AI-driven imaging biomarkers.

## 4. Discussion

Our investigation demonstrates that perivascular inflammation and morphologically HRP can be present even in patients with low or zero CACs. Despite minimal or absent calcification, severe CAD may still exist, characterized by features associated with plaque vulnerability—such as LAP components, PR, and the NRS. The application of FAI analysis enables the identification of elevated perivascular inflammation surrounding such lesions, revealing biologically active, high-risk atherosclerotic plaques that are undetectable by calcium scoring alone. These findings underscore the clinical value of integrating anatomical and inflammatory imaging for a more comprehensive assessment of CAD, especially in symptomatic or intermediate-risk patients.

Two previously published cases from our cohort emphasize the limitations of calcium scoring in both symptomatic and asymptomatic patients. The first described three middle-aged men with exertional chest pain and CACs of zero, all of whom had severe, non-calcified LAD stenoses and elevated perivascular inflammation on FAI analysis [23]. The second involved a middle-aged woman with low CACs who experienced a STEMI prior to a scheduled intervention, despite the presence of HRP features and markedly elevated FAI values [24]. These cases highlight the diagnostic gap when relying solely on calcium scoring and support the added value of incorporating inflammation-sensitive imaging to detect high-risk, non-calcified coronary lesions.

These results reinforce the increasingly accepted viewpoint that while CAC scoring is beneficial for long-term risk stratification in asymptomatic individuals, it substantially underestimates early-stage, non-calcified, lipid-rich plaques—particularly in symptomatic or high-risk patient populations [25,26]. Indeed, histopathological studies indicate that the culprit plaques underlying ACS typically lack calcification and feature thin fibrous caps with large lipid-rich cores, making them undetectable through calcium-based imaging but readily identifiable using CCTA [27]. In our patient cohort, individuals even within the CACs = 0 category displayed several morphological signs indicative of high-risk plaques and elevated inflammation, aligning with previous observations that approximately 4–10% of patients with obstructive CAD may exhibit a CAC score of zero [28]. Collectively, these data advocate strongly for the role of CCTA not merely to evaluate luminal narrowing but also to assess plaque composition and the surrounding PVAT, which significantly influences plaque progression.

In this context, the CaRi-Heart^®^ platform represents a meaningful advancement in coronary risk imaging [14]. By leveraging AI-driven radiomic analysis of perivascular fat, CaRi-Heart^®^ provides age- and sex-adjusted FAI centile scores that reflect localized vascular inflammation—offering diagnostic insights not captured by traditional anatomical imaging. Unlike structural plaque metrics or luminal assessments, FAI quantifies biologically active processes, which may precede visible calcification or stenosis. Prior studies, including CRISP-CT and ORFAN, have shown that elevated FAI scores independently predict adverse cardiovascular outcomes—even in the absence of obstructive CAD or high CACs [22,29,30]. Our study builds on this by applying CaRi-Heart^®^ in a real-world, low-to-intermediate risk population, stratified by CAC burden. We demonstrate that FAI can uncover inflammation-prone lesions in individuals who might otherwise be classified as low risk based solely on calcium scoring, enhancing early detection and risk stratification [31]. Notably, FAI centiles above 90 have been associated with a significantly increased risk of adverse events in prior prospective studies like CRISP-CT, although standardized thresholds for clinical reclassification or therapeutic intervention are not yet universally adopted.

FAI mapping, as integrated within CCTA analysis, further refines diagnostic precision by capturing vessel-specific inflammatory signals that systemic biomarkers like high-sensitivity C-reactive protein (hsCRP) cannot localize [32]. Studies by Goeller et al. and Simantiris et al. have demonstrated consistent links between inflamed PVAT, high-risk plaque (HRP) features, and rapid atherosclerotic progression [33,34]. Notably, perivascular inflammation may persist even in the absence of severe stenosis or dense calcification—highlighting the dissociation between plaque burden and vulnerability. Our findings reinforce this, illustrating that patients with minimal or no calcification may still harbor dangerous, inflamed plaques. Similar trends have been observed in post-COVID-19 populations, where persistent coronary inflammation was noted despite stable anatomical profiles [35,36,37].

In individuals with detectable coronary calcification (CAC > 0), increasing calcific burden correlated with both anatomical disease severity and localized inflammation. Specifically, higher CACs were associated with more extensive coronary involvement and elevated CAD-RADS categories. Regression analysis revealed a moderate correlation between CACs and the number of diseased coronary segments (r = 0.2520, *p* < 0.0001), and a weaker correlation with CAD-RADS categories (r = 0.1352, *p* < 0.0001). While CACs also correlated with the perivascular FAI score centile (r^2^ = 0.0568, *p* = 0.0003), this association was relatively weak—emphasizing the fact that FAI captures complementary, not overlapping, information relative to calcium burden. We acknowledge that modest correlations must be interpreted with caution; however, they may still signal residual inflammatory risk in patients with low or intermediate CACs. To avoid potential overdiagnosis or overtreatment, further longitudinal validation is warranted before implementing FAI-guided decision-making in routine practice. These data underscore the utility of combining anatomical and inflammatory imaging to better characterize atherosclerotic activity. While CACs reflect total plaque burden, FAI measures vascular inflammation—a key driver of instability [11,29]. Prior studies, including CRISP-CT, have confirmed FAI’s prognostic value even in patients with low or zero CACs [29,32,33], and inflamed PVAT has been linked to both HRP features and accelerated plaque evolution [38,39]. This supports the integration of inflammatory imaging biomarkers like FAI into routine CCTA interpretation, particularly in patients with minimal calcification [40,41].

## 5. Conclusions

This study underscores the limitations of CACs as a standalone tool for risk assessment in patients with suspected CAD. Our findings reveal that morphologically vulnerable and inflamed plaques may be present even in individuals with low or zero CACs—lesions that are overlooked by calcium scoring alone. By combining CCTA with perivascular FAI mapping, we were able to detect biologically active atherosclerotic disease with greater precision. This integrated anatomical and inflammatory approach enhances risk stratification and supports more informed clinical decision-making. Further prospective studies are needed to validate the prognostic value of FAI and define its role in routine cardiovascular evaluation.

## Figures and Tables

**Table 1:** Baseline clinical characteristics, cardiovascular risk factors, and SCORE2 risk classification stratified by CAC categories.

Parameters	Whole Study Sample (*n* = 250)	Group 1 (CACs = 0) (*n* = 28)	Group 2 (CACs 1–100) (*n* = 121)	Group 3 (CACs > 100) (*n* = 101)	*p* Value ^1^	*p* Value ^2^	*p* Value ^3^	*p* Value ^4^
Age, (years), mean ± SD	61.1 ± 6.3	55.9 ± 7.1	60.4 ± 6.4	63.4 ± 4.8	0.0011	0.0004	<0.0001	<0.0001
Male gender, *n* (%)	185 (74.00%)	17 (60.71%)	88 (72.73%)	80 (79.21%)	0.7540	0.8284	0.1561	0.1290
BMI ^5^, (kg/m^2^), mean ± SD	27.67 ± 3.8	25.05 ± 3.5	27.93 ± 3.9	28.07 ± 3.4	0.0008	0.9589	0.0005	0.0005
LVEF ^6^ (%), mean ± SD	54.43 ± 4.55	54.46 ± 4.53	54.17 ± 4.07	54.73 ± 5.09	0.9504	0.6349	0.9590	0.6613
Cardiovascular risk factors:								
Hypertension, *n* (%)	150 (60.00%)	9 (32.14%)	71 (58.68%)	70 (69.31%)	0.0376	0.3710	0.0023	0.0017
Hypercholesterolemia, *n* (%)	142 (56.80%)	7 (25.00%)	69 (57.02%)	66 (65.35%)	0.0089	0.6513	0.0006	0.0007
Smoking, *n* (%)	71 (28.40%)	5 (17.86%)	38 (31.40%)	28 (27.72%)	0.5203	>0.9999	>0.9999	0.3515
Obesity, *n* (%)	51 (20.40%)	3 (10.71%)	28 (23.14%)	20 (19.80%)	0.5941	>0.9999	>0.9999	0.3329
Familial history of CAD ^7^, *n* (%)	44 (17.60%)	2 (7.14%)	22 (18.18%)	22 (21.78%)	0.7551	>0.9999	0.3033	0.2084
SCORE2 ^8^ risk assessment:								
Low–moderate risk, *n* (%)	103 (41.20%)	16 (57.14%)	47 (38.84%)	40 (39.60%)	0.2754	>0.9999	0.3939	0.1901
High risk, *n* (%)	87 (34.80%)	8 (28.57%)	45 (37.19%)	34 (33.66%)	>0.9999	>0.9999	>0.9999	0.6567
Very high risk, *n* (%)	60 (24.00%)	4 (14.29%)	29 (23.97%)	27 (26.73%)	0.9654	>0.9999	0.6498	0.3941

^1^ Group 1 vs. Group 2; ^2^ Group 2 vs. Group 3; ^3^ Group 1 vs. Group 3; ^4^ Overall comparison via one-way ANOVA (for continuous variables, with Tukey’s HSD post hoc test) or Chi-square test (for categorical variables, with Fisher’s exact test and Bonferroni correction applied to pairwise comparisons; α = 0.0167); ^5^ BMI—body mass index; ^6^ LVEF—left ventricular ejection fraction; ^7^ CAD—coronary artery disease; ^8^ SCORE2—Systematic Coronary Risk Evaluation 2.

**Table 2:** Summary of plaque composition, HRP characteristics, and CaRi-Heart^®^ inflammation-based risk classification stratified by CAC categories.

Parameters	Whole Study Sample (*n* = 250)	Group 1 (CACs = 0) (*n* = 28)	Group 2 (CACs 1–100) (*n* = 121)	Group 3 (CACs > 100) (*n* = 101)	*p* Value ^1^	*p* Value ^2^	*p* Value ^3^	*p* Value ^4^
Segments/Subject, Mean ± SD, (Range), {Sum}	3.18 ± 1.93 (0–8), {797}	1.39 ± 1.10 (0–4), {39}	2.97 ± 1.57 (1–6), {360}	3.94 ± 2.10 (1–8), {398}	<0.0001	0.0002	<0.0001	<0.0001
Segment involvement score:								
≤2, *n* (%)	107 (42.80%)	23 (82.14%)	55 (45.45%)	29 (28.71%)	0.0018	0.0372	<0.0001	<0.0001
3–4, *n* (%)	78 (31.20%)	5 (17.86%)	38 (31.40%)	35 (34.65%)	0.5203	>0.9999	0.3257	0.2362
>4, *n* (%)	65 (26.00%)	0 (0.00%)	28 (23.14%)	37 (36.63%)	0.0070	0.1132	<0.0001	0.0002
Plaque composition:								
Non-calcified plaques, *n* (%)	133 (16.69%)	39 (100.00%)	82 (22.78%)	12 (3.02%)	<0.0001	<0.0001	<0.0001	<0.0001
Mixed plaques, *n* (%)	324 (40.65%)	0 (0.00%)	168 (46.67%)	156 (39.20%)	<0.0001	>0.9999	<0.0001	<0.0001
Calcified plaques, *n* (%)	340 (42.66%)	0 (0.00%)	110 (30.56%)	230 (57.79%)	<0.0001	<0.0001	<0.0001	<0.0001
HRP ^5^ characteristics:								
HRP feature prevalence, *n* (%)	371 (46.55%)	5 (12.82%)	104 (28.89%)	262 (65.83%)	0.1098	<0.0001	<0.0001	<0.0001
PR ^6^, *n* (%)	89 (11.17%)	2 (5.13%)	36 (10.00%)	51 (12.81%)	>0.9999	0.7626	>0.9999	0.2213
LAP ^7^, *n* (%)	71 (8.91%)	3 (7.69%)	18 (5.00%)	50 (12.56%)	>0.9999	0.0010	0.0004	0.0012
SC ^8^, *n* (%)	158 (19.82%)	0 (0.00%)	37 (10.28%)	121 (30.40%)	0.1107	<0.0001	<0.0001	<0.0001
NRS ^9^, *n* (%)	53 (6.65%)	0 (0.00%)	13 (3.61%)	40 (10.05%)	>0.9999	0.0016	0.0373	0.0004
CAD-RADS ^10^ categories:								
0–1, *n* (%)	105 (42.00%)	20 (71.43%)	70 (57.85%)	15 (14.58%)	0.6170	<0.0001	<0.0001	<0.0001
2–3, *n* (%)	78 (31.20%)	5 (17.86%)	37 (30.58%)	36 (35.64%)	0.7336	>0.9999	0.3218	0.1946
4–5, *n* (%)	67 (26.80%)	3 (10.71%)	14 (11.57%)	50 (49.50%)	>0.9999	<0.0001	0.0004	<0.0001
CaRi-Heart^®^ risk assessment:								
Low risk, *n* (%)	117 (46.80%)	19 (67.86%)	60 (49.59%)	38 (37.62%)	0.2854	0.2379	0.0160	0.0124
Medium risk, *n* (%)	67 (26.80%)	6 (21.43%)	32 (26.45%)	29 (28.71%)	>0.9999	>0.9999	>0.9999	0.7379
High risk, *n* (%)	66 (26.40%)	3 (10.71%)	29 (23.97%)	34 (33.66%)	0.5988	0.4051	0.0550	0.0358

^1^ Group 1 vs. Group 2; ^2^ Group 2 vs. Group 3; ^3^ Group 1 vs. Group 3; ^4^ Overall comparison via one-way ANOVA (for continuous variables, with Tukey’s HSD post hoc test) or Chi-square test (for categorical variables, with Fisher’s exact test and Bonferroni correction applied to pairwise comparisons; α = 0.0167); ^5^ HRP—high-risk plaque; ^6^ PR—positive remodeling; ^7^ LAP—low-attenuation plaque; ^8^ SC—spotty calcification; ^9^ NRS—napkin-ring sign; ^10^ CAD-RAD—Coronary Artery Disease-Reporting and Data System.

## Data Availability

The data presented in this study are available on request from the corresponding author. The data are not publicly available due to privacy reasons.

## Abbreviations

The following abbreviations are used in this manuscript:

ACS	Acute Coronary Syndrome
AI	Artificial Intelligence
BMI	Body Mass Index
CABG	Coronary Artery Bypass Grafting
CACs	Coronary Artery Calcium Score
CAD	Coronary Artery Disease
CAD-RADS	Coronary Artery Disease-Reporting and Data System
CaRi-Heart^®^	AI-powered Platform for Coronary Risk Prediction
CCTA	Coronary Computed Tomography Angiography
CVD	Cardiovascular Disease
DM	Diabetes Mellitus
DICOM	Digital Imaging and Communications in Medicine
ESC	European Society of Cardiology
FAI	Fat Attenuation Index
HDL-Cho	High-Density Lipoprotein Cholesterol
HRP	High-Risk Plaque
hsCRP	High-Sensitivity C-Reactive Protein
MI	Myocardial Infarction
LAD	Left Anterior Descending artery
LAP	Low-Attenuation Plaque/Core
LCX	Left Circumflex Artery
LDL-Cho	Low-Density Lipoprotein Cholesterol
LVEF	Left Ventricular Ejection Fraction
MACE	Major Adverse Cardiovascular Events
NRS	Napkin-Ring Sign
PCI	Percutaneous Coronary Intervention
PR	Positive Remodeling
PVAT	Perivascular Adipose Tissue
RCA	Right Coronary Artery
SC	Spotty Calcification
SIS	Segment Involvement Score
STEMI	ST-Elevation Myocardial Infarction
TGs	Triglycerides
T-Cho	Total Cholesterol
